# MTOR PROMOTES BBB BREAKDOWN IN A MODEL OF ALZHEIMER’S DISEASE

**DOI:** 10.1093/geroni/igz038.957

**Published:** 2019-11-08

**Authors:** Stephen F Hernandez, Candice E Van Skike, Nick DeRosa, Veronica Galvan

**Affiliations:** 1 UT Health San Antonio, San Antonio, Texas, United States; 2 Barshop Institute - UT Health San Antonio, San Antonio, Texas, United States; 3 Barshop Institute for Longevity and Aging Studies, San Antonio, Texas, United States

## Abstract

Cerebral amyloid angiopathy (CAA) is characterized by fibrillar amyloid β (Aβ) association with cerebrovasculature, which leads to impaired brain vascular function, and is present in 87% of people with Alzheimer’s disease (AD). We previously showed that inhibition of mTOR by rapamycin prevented BBB breakdown and reduced vascular fibrillar Aβ in 18-19 month old Tg2576 mice that model AD-associated CAA. This finding suggests that mTOR attenuation restores integrity of the blood brain barrier (BBB) and concomitantly reduces vascular Aβ accumulation in this mouse model. Objective: To determine the mechanisms by which mTOR drives BBB breakdown we measured the abundance of tight junction proteins zonula occludens 1 (ZO-1), occludin, and claudin-5. Methods: We used immunofluorescent confocal microscopy on frozen brain tissue sections of the same Tg2576 mice used in the previous study. Results: We confirm BBB breakdown in Tg2576 mouse brains and showed that some, but not all tight junction proteins measured were decreased in cerebrovasculature of Tg2576 mice. Attenuation of mTOR by rapamycin preserved BBB integrity, decreased vascular Aβ accumulation, and increased levels of tight junction protein abundance in Tg2576 mice, which also showed a reduced numbers of cerebral microhemorrhages. Conclusions: Taken together, these data suggest that mTOR promotes brain vascular Aβ deposition, BBB breakdown and vascular damage in the Tg2576 mouse model. Thus, mTOR inhibitors such as rapamycin – an FDA approved drug - may have promise in the treatment of AD and other dementias with related cerebrovascular dysfunction.

